# Assessment of the immune-modulatory activity of sialylated fraction of IVIg in a murine model of allergic asthma

**DOI:** 10.1186/1710-1492-7-S2-A29

**Published:** 2011-11-14

**Authors:** Amir H Massoud, Aidan Ablona, Walid Mourad, Ciriaco Piccirillo, Bruce Mazer

**Affiliations:** 1 Meakins-Christie Laboratories, McGill University, Montreal QC. Canada H2X 2P2; 2 Département d’Immunologie et Microbiologie, Université de Montréal, Montreal, QC. Canada H3C 3J7; 3 Department of Microbiology and Immunology, McGill University Montreal, QC. Canada H3G 1A4

## Background

Intravenous immunoglobulin (IVIg) has potent immune-modulating properties. In OVA-challenged mice, we demonstrated that IVIg markedly attenuates airway hyperresponsiveness (AHR) and abrogates airways inflammation, accompanied by substantial induction of antigen-specific Foxp3^+^ Treg from non-Treg precursors.

## Methods

Mice were sensitized (i.n.) with OVA and then received IVIg or sialic acid enriched IVIg (SA-IVIg) fragments (i.p.), and then underwent challenge (i.n.). The induction of CD4^+^CD25^+^Foxp3^+^Treg was determined by flow-cytometry. AHR was measured, using a flexiVent small animal ventilator. Phenotypic properties of dendritic cells (DC) from various experimental groups were assessed by flow-cytometry. Expression of DCIR on DC was evaluated by flowcytometry and ICC. Adoptive transfer of DC was carried out to show the tolerogenic activity of IVIg-primed DC.

## Results

IVIg and the SA-IVIg fraction induced Treg and abrogated AHR in OVA-challenged mice comparably. It followed by tolerogenic predisposition of DC (decrease of CD80/CD86 expression and IFN-γ production and increased level of IL-10). Adoptive transfer of DC from IVIg treated mice to OVA-challenged WT syngeneic mice has the similar anti-inflammatory activity of IVIg/SA-IVIg. Expression of DCIR (Inhibitory C-type lectin receptors) on DC of IVIg and SA-IVIg treated mice increased significantly.

## Conclusions

IVIg induces Treg likely via conferring tolerogenic activities to DC. This mechanism is dependent on sialylated fraction of IVIg. DCIR is an inhibitory C-type lectin receptor that can be targeted by SA-IVIg and induce an inhibitory signal into the ligated cells. More dissection is required to confirming the role of DCIR in this model.

